# Lead Abandonment and Subcutaneous Implantable Cardioverter-Defibrillator (S-ICD) Implantation in a Cohort of Patients With ICD Lead Malfunction

**DOI:** 10.3389/fcvm.2021.692943

**Published:** 2021-07-27

**Authors:** Vincenzo Russo, Stefano Viani, Federico Migliore, Gerardo Nigro, Mauro Biffi, Gianfranco Tola, Giovanni Bisignani, Antonio Dello Russo, Paolo Sartori, Roberto Rordorf, Luca Ottaviano, Giovanni Battista Perego, Luca Checchi, Luca Segreti, Emanuele Bertaglia, Mariolina Lovecchio, Sergio Valsecchi, Maria Grazia Bongiorni

**Affiliations:** ^1^Department of Medical Translational Sciences, University of Campania “Luigi Vanvitelli”, Monaldi Hospital, Naples, Italy; ^2^Second Cardiology Division, Cardio-Thoracic and Vascular Department, University Hospital of Pisa, Pisa, Italy; ^3^Department of Cardiac, Thoracic and Vascular Sciences, University of Padova, Padova, Italy; ^4^Institute of Cardiology, University of Bologna, Policlinico S.Orsola-Malpighi, Bologna, Italy; ^5^Cardiology Division, A.O. Brotzu, Cagliari, Italy; ^6^Department of Cardiology, Ospedale “Ferrari”, Cosenza, Italy; ^7^Clinica di Cardiologia e Aritmologia, Università Politecnica delle Marche, Ancona, Italy; ^8^Cardiology Division, Hospital IRCCS San Martino, Genoa, Italy; ^9^Department of Cardiology, Fondazione IRCCS Policlinico S. Matteo, Pavia, Italy; ^10^Cardiology Division, Istituto Clinico S. Ambrogio, Milan, Italy; ^11^Cardiology Division, Istituto Auxologico Italiano, Milan, Italy; ^12^Cardiology Division, University of Florence, Florence, Italy; ^13^Rhythm Management Department, Boston Scientific, Milan, Italy

**Keywords:** implantable defibrillator, subcutaneous, lead extraction, lead abandonment, lead malfunction

## Abstract

**Background:** When an implantable-cardioverter defibrillator (ICD) lead becomes non-functional, a recommendation currently exists for either lead abandonment or removal. Lead abandonment and subcutaneous ICD (S-ICD) implantation may represent an additional option for patients who do not require pacing. The aim of this study was to investigate the outcomes of a strategy of lead abandonment and S-ICD implantation in the setting of lead malfunction.

**Methods:** We analyzed all consecutive patients who underwent S-ICD implantation after abandonment of malfunctioning leads and compared their outcomes with those of patients who underwent extraction and subsequent reimplantation of a single-chamber transvenous ICD (T-ICD).

**Results:** Forty-three patients underwent S-ICD implantation after abandonment of malfunctioning leads, while 62 patients underwent extraction and subsequent reimplantation of a new T-ICD. The two groups were comparable. In the extraction group, no major complications occurred during extraction, while the procedure failed and an S-ICD was implanted in 4 patients. During a median follow-up of 21 months, 3 major complications or deaths occurred in the S-ICD group and 11 in the T-ICD group (HR 1.07; 95% CI 0.29–3.94; *P* = 0.912). Minor complications were 4 in the S-ICD group and 5 in the T-ICD group (HR 2.13; 95% CI 0.49–9.24; *P* = 0.238).

**Conclusions:** In the event of ICD lead malfunction, extraction avoids the potential long-term risks of abandoned leads. Nonetheless the strategy of lead abandonment and S-ICD implantation was feasible and safe, with no significant increase in adverse outcomes, and may represent an option in selected clinical settings. Further studies are needed to fully understand the potential risks of lead abandonment.

**Clinical Trial Registration:** URL: ClinicalTrials.gov Identifier: NCT02275637

## Introduction

Implantable cardioverter-defibrillators (ICDs) are an effective therapy for sudden cardiac death prevention (1). However, complications with ICD therapy exist and are mainly associated with the use of transvenous leads in the heart and vascular system (2, 3). In the case of lead malfunction, it may be removed or left *in situ*, and the decision should be based on the expected risks and benefits (4). The risks of removal include venous or cardiac perforation, and depend on many factors, such as the duration of the lead implant, the patient's age and condition, and the experience of the operator. The benefits of removal include the avoidance of possible infections requiring later and more difficult extraction, and the creation of an access to allow implantation of a new lead. Currently, in the setting of lead malfunction, a class IIa recommendation exists for either lead abandonment or removal (4); this is based on single-center observational studies that have compared the two strategies, followed by transvenous ICD (T-ICD) reimplantation (5, 6). An entirely subcutaneous ICD (S-ICD) (Emblem™, Boston Scientific Inc., Natick, MA, USA) has been developed to prevent all possible complications associated with the insertion and long-term presence of transvenous leads in the heart and the vascular system (7, 8). Since with S-ICD no leads are inserted into the cardiovascular system, it may represent a preferred option for patients with limited venous access or those who are at high risk of infection (9). Thus, a strategy of S-ICD implantation after the abandonment of malfunctioning leads may represent an additional option for patients who do not require pacing.

The aim of the present study was to compare outcomes of a strategy of lead abandonment and S-ICD implantation in the setting of lead malfunction, with those of patients who underwent transvenous extraction with subsequent reimplantation of a single-chamber T-ICD.

## Methods

### Study Design

Patients undergoing implantation of an ICD were prospectively enrolled at the cardiovascular centers that participate in the Rhythm Detect registry (NCT02275637). The Institutional Review Boards approved the study, and all patients provided written informed consent for data storage and analysis. For the present analysis, we identified all patients, from 2015 to 2018 at 12 Italian centers, who underwent S-ICD (Boston Scientific Inc., Natick, MA, USA) implantation after the abandonment of malfunctioning leads and compared their outcomes with those of patients who underwent transvenous extraction with subsequent reimplantation of a single-chamber T-ICD. Baseline assessment comprised collection of demographic data, medical history (including data from the extraction and subsequent implantation procedures), clinical examination, 12-lead electrocardiogram, echocardiography and estimation of NYHA functional class. The extraction and implantation procedures, as well as perioperative and postoperative clinical management, were performed in accordance with the clinical practice of each center. In patients who received an S-ICD, an ECG morphology tool was used to verify the quality of device sensing before implantation. Devices were implanted and acute defibrillation tests were performed according to the local clinical practice. Defibrillation testing through induction of ventricular fibrillation was performed under deep sedation or general anesthesia. Information on clinical outcomes, such as hospitalizations and deaths, was collected during hospital visits or, if patients missed scheduled visits, via telephone calls.

### Study End-Points

In the present analysis, the study database was searched for all procedure- or device-related adverse events, defined as untoward events resulting from the presence or performance of the system implanted. Specifically, those events resulting in prolonged hospitalization or surgical intervention for system revision were considered to be major complications. The primary endpoint was the combination of major complications and all-cause deaths. All adverse events not requiring surgical intervention (including inappropriate shocks) or hospitalization were classified as minor complications. The end-points were analyzed according to the intention-to-treat principle. On-treatment analysis was also performed.

### Statistical Analysis

Descriptive statistics are reported as means ± SD for normally distributed continuous variables or medians with 25th to 75th percentiles in the case of skewed distribution. Normality of distribution was tested by means of the non-parametric Kolmogorov-Smirnov test. Categorical variables are reported as percentages. Differences between mean data were compared by means of a t-test for Gaussian variables, and Mann–Whitney non-parametric test for non-Gaussian variables. Differences in proportions were compared by means of chi-square analysis or Fisher's exact test, as appropriate. Survival analysis was performed by means of the Kaplan-Meier method, and the log-rank test was applied to evaluate differences between survival trends. For all time-to-event estimations, patients were censored on death or at their last follow-up visit. A *P* value < 0.05 was considered significant for all tests. All statistical analyses were performed by means of STATISTICA software, version 7.1 (StatSoft, Inc, Tulsa, OK, USA).

## Results

### Study Population

From 2015 to 2018, a total of 43 patients underwent S-ICD implantation after the abandonment of malfunctioning leads at the study centers. In the same period, transvenous extraction of malfunctioning leads and subsequent reimplantation of a single-chamber T-ICD was attempted in 62 patients. Table 1 shows the baseline clinical variables in the two groups. Age, left ventricular systolic function, functional status and etiology were comparable between the groups. Chronic kidney disease was non-significantly more frequent in patients with abandoned leads and S-ICD (*p* = 0.054). A multi-lead (dual-chamber or biventricular) ICD had previously been implanted in 33% of patients who subsequently received an S-ICD and in 21% of those who underwent extraction and received a single-chamber ICD (*p* = 0.181, Table 1). The implant duration (time from the first ICD implantation) was significantly longer in patients with lead abandonment and S-ICD implantation (5 ± 3 vs. 4 ± 2 years, *p* = 0.021).

**Table 1:** Demographics and baseline clinical parameters.

**Parameter**	**All patients**	**Lead abandonment and S-ICD**	**Lead extraction and T-ICD**	***p*-value**
	***n* = 105**	***n* = 43**	***n* = 62**	
Male gender, *n* (%)	80 (76)	36 (84)	44 (71)	0.131
Age, years	55 ± 17	55 ± 16	54 ± 18	0.749
Body Mass Index	25 ± 5	26 ± 4	24 ± 5	0.084
LV ejection fraction, %	46 ± 15	43 ± 15	48 ± 14	0.096
NYHA Class III-IV, *n* (%)	13 (12)	6 (14)	7 (11)	0.684
Ischemic/Non-ischemic	60 (57)	27 (63)	33 (53)	0.330
Cardiomyopathy, *n* (%)				
Hypertrophic Cardiomyopathy, *n* (%)	9 (9)	3 (7)	6 (10)	0.734
Congenital/ARVD, *n* (%)	13 (12)	5 (12)	8 (13)	0.845
Channelopathies/Other, *n* (%)	23 (22)	8 (18)	15 (24)	0.496
Chronic kidney disease, *n* (%)	20 (19)	12 (28)	8 (13)	0.054
Diabetes, *n* (%)	15 (14)	6 (14)	9 (15)	0.974
Previous dual-chamber ICD, *n* (%)	24 (23)	13 (30)	11 (18)	0.133
Previous biventricular ICD, *n* (%)	3 (3)	1 (2)	2 (3)	1.000
Number of previous leads,	1.3 ± 0.5	1.3 ± 0.5	1.2 ± 0.5	0.249
Time from first implant, years	4 ± 3	5 ± 3	4 ± 2	0.021

*NYHA, New York Heart Association; LV, Left ventricular; ARVD, Arrhythmogenic Right Ventricular Dysplasia; ICD, Implantable Cardioverter Defibrillator*.

### S-ICD Implantation Procedure

The surface ECG screening procedure identified at least 1 suitable vector in all patients; at least two vectors were appropriate in 37 (86%), and three vectors in 15 (35%). The S-ICD generator was positioned in a standard subcutaneous pocket in 8 (19%) patients, while an intermuscular approach was adopted in the remaining 81%. The lead was positioned by means of a 2-incision technique (avoiding the superior parasternal incision) in 38 (88%) patients. Defibrillation testing was performed in 31 S-ICD patients (72%) and was effective in all cases at 80J and in 30 (96.7%) cases at 65J. In the remaining patients, defibrillation testing was not performed because of concerns over reported clinical instability (3 patients), lack of inducibility of ventricular fibrillation (3 patients), or physician preference (6 patients). On hospital discharge, sensing from the primary vector was programmed in 26 (60%) patients, from the secondary vector in 15 (35%) and from the alternative vector in 2 (5%).

### Lead Extraction and Transvenous ICD Implantation

A total of 75 leads (1.2 ± 0.5 leads per patient) were extracted from 62 patients. Leads were extracted by means of locking stylets in 1 (2%) patient, mechanical non-powered sheaths (Byrd Dilator Polypropylene Sheaths©, Cook Medical, Bloomington, IN, USA) in 57 (92%) and powered sheaths (multiple manufacturers) in 4 (6%). Complete procedural success was obtained in 56 (90%) patients. Partial success (<4 cm lead fragment remained in the body) was reported in 1 (2%) patient and radiological failure (>4 cm lead fragment remained in the body) was reported in 5 (8%) patients. In 4 of these 5 patients, the decision was taken to implant an S-ICD; these 4 patients were analyzed in the lead extraction group, according to the intention-to-treat principle, and in the lead abandonment and S-ICD group, according to the on-treatment principle. No major complications occurred during extraction. Pocket hematomas were reported in two patients. In one case, it resolved without specific therapy; in the other, it required evacuation and was associated with a vagal crisis rapidly resolved with fluid infusion and atropine.

### Follow-Up

**Figure 1:**
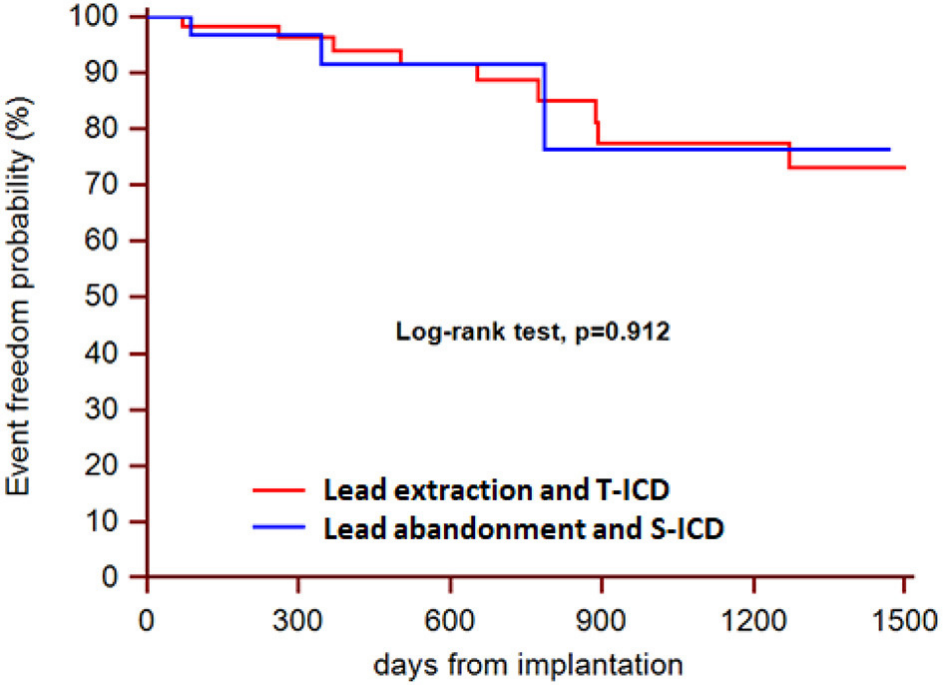
Kaplan-Meier estimates of time to the primary endpoint, according to intention-to-treat principle.

**Figure 2:**
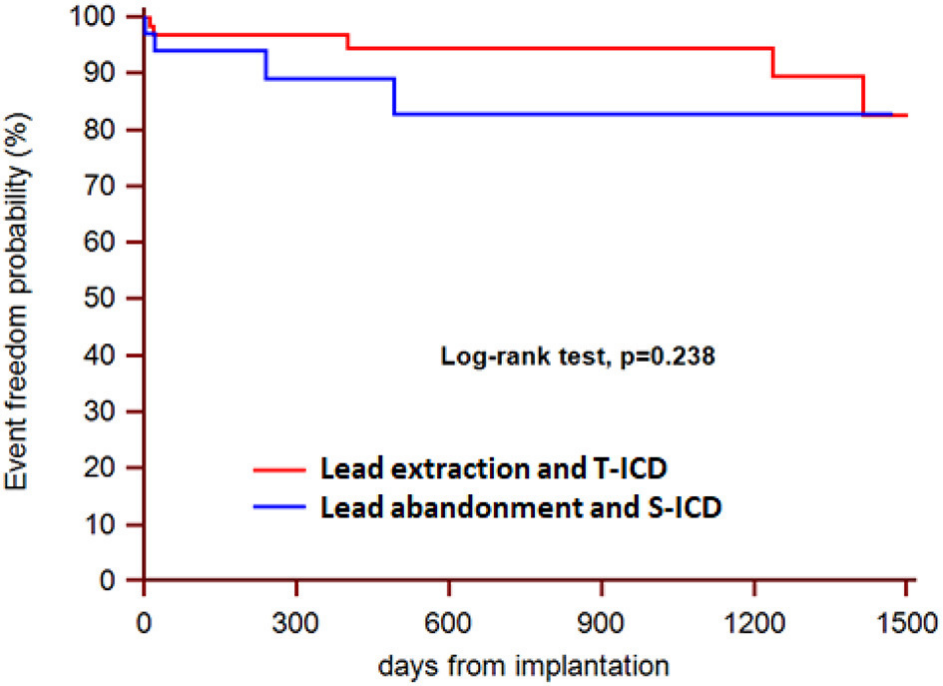
Kaplan-Meier estimates of time to first minor complication, according to intention-to-treat principle.

During a median follow-up of 21 months (25th to75th percentiles, 7 to 39), 3 patients died (1 in the S-ICD group and 1 in the T-ICD group from chronic heart failure, and 1 in the T-ICD group from non-cardiac reasons). One patient with T-ICD underwent urgent heart transplantation. No sudden cardiac deaths occurred. Additional major complications were reported in 10 patients (Table 2). All complications were successfully resolved. Moreover, 9 additional events were managed non-invasively, and were defined as minor complications (Table 3). The Kaplan-Meier estimates of time to the primary endpoint were compared between the groups according to the intention-to-treat principle (hazard ratio, 1.07; 95% CI, 0.29 to 3.94; *p* = 0.912) (Figure 1). Similar findings were obtained with the analysis of time to first major complication (hazard ratio, 0.85; 95% CI, 0.19 to 3.74; *p* = 0.834). In the on-treatment analysis, estimates of time to the primary endpoint were compared between the 47 patients who actually had abandoned leads and received S-ICD and the 58 patients who underwent successful lead extraction and T-ICD implantation (hazard ratio, 1.35; 95% CI, 0.39 to 4.69; *p* = 0.590). The Kaplan-Meier analysis of minor complications according to the intention-to-treat principle is reported in Figure 2 (hazard ratio, 2.13; 95% CI, 0.49 to 9.24; *p* = 0.238). The on-treatment analysis yielded similar findings (hazard ratio, 1.79; 95% CI, 0.44 to 7.36; *p* = 0.365).

**Table 2:** Major complications reported during follow-up.

**Major complications**	**Number**	**Resolution**	**Details**
***Lead abandonment and S-ICD***			
Early depletion	1	Device replacement	
Need for bradycardia pacing	1	Leadless pacemaker implantation	*Atrioventricular block*
***Lead extraction and T-ICD***			
Early depletion	2	Device replacement (2)	
Surgical revision	1	Extraction	*Previous extraction failure*
Lead dislodgement	1	Lead repositioning	
Need for resynchronization therapy	3	System upgrade (3)	*The previous ICDs were single- (2) and dual-chamber ICD (1)*
Systemic infection	1	Resolved with in-hospital antibiotic therapy	

**Table 3:** Minor complications reported during follow-up.

**Minor complications**	**Number**	**Resolution**	**Details**
***Lead abandonment and S-ICD***			
Pocket hematoma	2	Resolved with no specific therapy (1)	
		Hematoma requiring evacuation (1)	
Inappropriate shock	2	Device reprogramming (2)	
***Lead extraction and T-ICD***			
Pocket hematoma	2	Resolved with no specific therapy (2)	
Inappropriate shock	2	Device reprogramming (2)	
Ineffective therapy	1	Device reprogramming	*Hemodynamically stable ventricular tachycardia accelerated into fibrillation*

## Discussion

Despite the proven effectiveness of ICD therapy in preventing sudden cardiac death, the transvenous lead still constitutes the weakest link in the chain. According to the literature, the annual rate of transvenous ICD lead failure may reach 20% in 10-year-old leads (10), and in recent years an unexpectedly high failure rate, related to structural issues, has been reported for some specific lead types (11, 12).

In the event of lead failure, either extraction and reimplantation or abandonment and the addition of a new lead may be considered. The advantages and disadvantages of both strategies need to be weighed carefully. In patients who do not require pacing, a third possible solution could be to implant an S-ICD and to leave the malfunctioning T-ICD lead in place. This approach avoids the risks of lead removal and those related to the insertion of additional transvenous leads. In our experience, the strategy of lead abandonment and S-ICD implantation appeared to be feasible and safe, with no increase in adverse outcomes.

Although the two groups in analysis were similar, the lead abandonment and S-ICD implantation strategy seemed to be preferred by the study centers in patients with a higher risk profile, i.e. with comorbidities, such as chronic kidney disease, with longer implant durations and with more leads in place. This is in line with the results of a European survey that investigated operators' views on the management of malfunctioning leads (13). Indeed, the variables associated with the decision to extract or abandon a lead included the lead-dwelling time and the total number of leads.

The S-ICD implantation procedure was found to be safe, with no complications reported, in agreement with previous and larger reports on *de-novo* S-ICD implantation (7, 8, 14). As previously described with regard to *de-novo* S-ICD implantation procedures in Europe (15), in most of our patients the generator was positioned in an intermuscular pocket and the lead was implanted by means of a 2-incision technique (16). Our findings extend those of our previous study on the use of S-ICD in patients undergoing ICD extraction, in which we recorded a reduction in complications when intermuscular generator positioning was adopted (17). Defibrillation testing was effective at 65J in 96.7% and at 80 J in 100% of patients; comparable success rates have been reported for *de-novo* implantation procedures (18). This confirms the effectiveness of the defibrillation wave generated by the S-ICD system, even in the presence of abandoned defibrillation coils.

In the present study, we also observed a high rate of success of the transvenous lead extraction procedure in the extraction/reimplantation group, with few radiological failures and only minor complications. These findings are in line with the results of the European Lead Extraction ConTRolled (ELECTRa) study, in which transvenous lead extraction generally proved safe and effective when performed at high-volume centers and by experienced operators (19). However, the procedure remains potentially associated with life-threatening operative and postoperative complications. Thus, the availability of an alternative approach may be extremely valuable, when extraction is not mandated in order to eradicate an infection. Interestingly, after a failed extraction attempt in 4 patients, the decision was taken to abandon the leads and to implant an S-ICD. Plausibly, the decision to interrupt the extraction procedure was prompted by the availability of an alternative solution.

In this analysis, the rates of complications during follow-up were comparable between the groups. This finding agrees with the results of studies that compared the performance of S-ICD and T-ICD after *de-novo* implantation (20) and with those of our previous study comparing S-ICD with T-ICD implantation after T-ICD explantation because of infection or for other reasons (17). The present analysis also complements a previous comparison between patients undergoing S-ICD implantation after extraction of a T-ICD and patients receiving a *de-novo* S-ICD (21), which documented similar complication rates.

Our results showed that there was no increased risk of complications, such as ineffective or inappropriate shocks or infections, related to abandoned leads. Although a recent publication (22) showed that 9% of abandoned ICD leads needed to be extracted at a median follow up of 4.4 ± 3.1 years, mostly due to infection. This is relevant not only in comparison with the patients who underwent extraction in this series, but also, and especially, if a strategy consisting of placement of an additional lead and T-ICD use is considered after lead abandonment (23). Indeed, Wollmann et al. (24) found a 3-year adverse event rate of 30% in patients who underwent implantation of an additional lead. An abandoned lead may interfere with the active transvenous lead and result in inappropriate shocks. Moreover, the presence of multiple leads is associated with higher risk of infection (25), and ICD-related infections carry significant risks of mortality and morbidity (26). Additional implications of lead abandonment and the placement of additional leads are the increased risk of venous thrombosis (27) and additional difficulties in the case of future extraction (28). Moreover, according to a recently published ELECTRa study sub-analysis, in the case of mandatory extraction (i.e. for infection), the presence of previously abandoned leads is associated with increased procedural complexity, clinical failure, and major complication rates (29). Our study revealed the practice of re-evaluating the need for pacing. Indeed, single-chamber ICDs and S-ICDs were frequently adopted after removal of dual-chamber or biventricular ICDs. Nevertheless, adopting this approach requires caution, in order to avoid the need for subsequent upgrades. Indeed, in our series, a leadless pacemaker was implanted in one S-ICD patient after verification of the need for bradycardia pacing. Similarly, the need for resynchronization therapy was identified during follow-up in 3 patients in the T-ICD group, and an additional procedure was required in order to upgrade the system.

**Figure 3:**
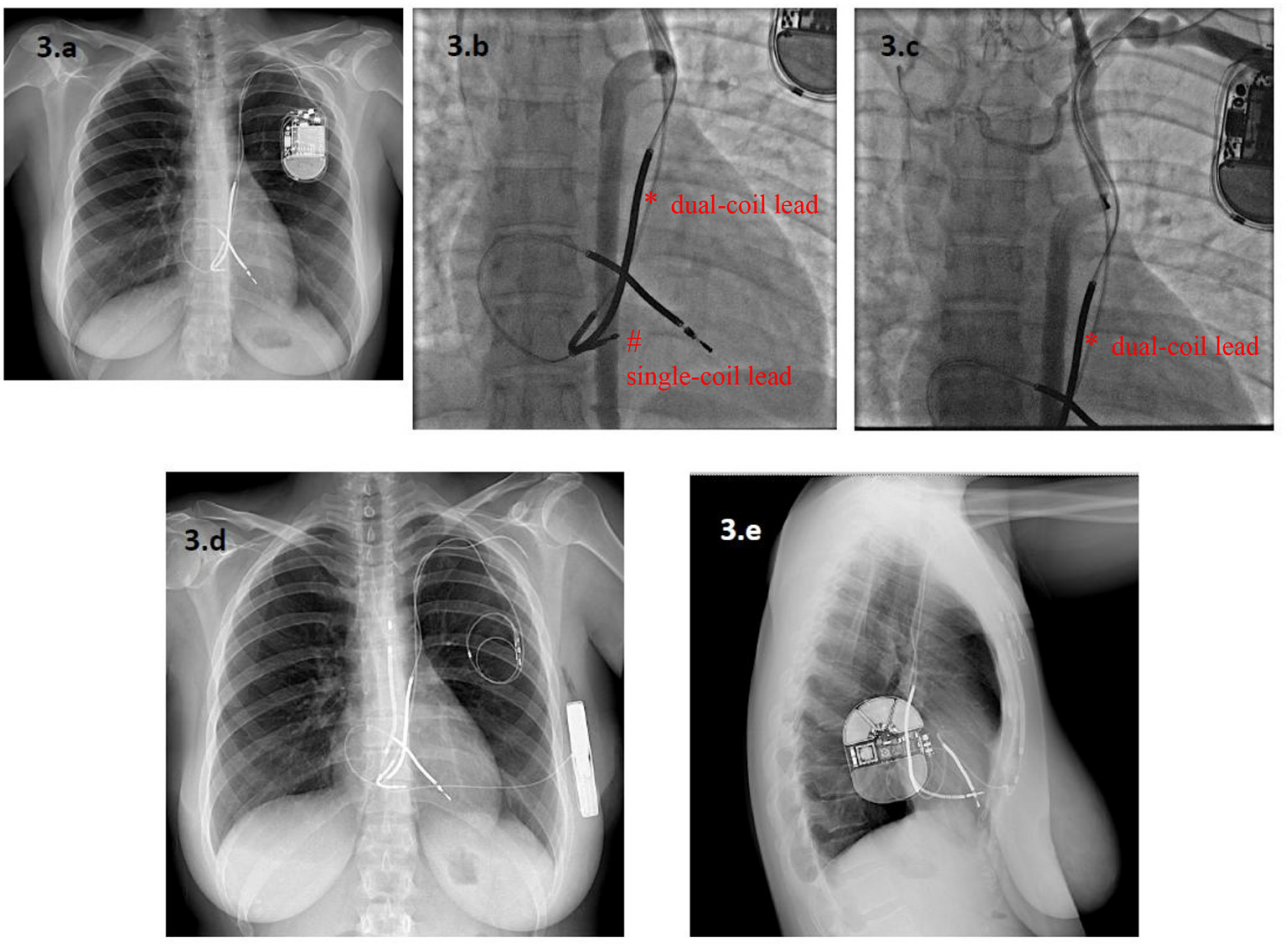
A 33-year-old woman with a Long QT Syndrome received a single-chamber T-ICD and a dual-coil lead via a persistent left superior vena cava (PLSVC) after a cardiac arrest. A new single-coil ICD lead was added **five** years later owing to malfunction of the first one, which was abandoned **(a)**. After seven years, the second ICD lead also malfunctioned. Angiography showed complete occlusion of the PLSVC, with a variant venous circulation from an accessory hemiazygos vein. The dual-coil ICD lead (*) and the single-coil lead (#) are visible in **(b,c)**. In this setting, lead extraction was considered to be at very high risk of venous laceration, while implantation of a new lead from the right side was deemed inappropriate because of the patient's young age. Finally, an S-ICD was implanted and both leads were abandoned **(d,e)**. * dual-coil lead # single-coil lead.

According to the data from the ELECTRa study (19), lead malfunction is becoming a more frequent indication for lead extraction than it was in the past (30). Currently, a class IIa recommendation exists for either lead abandonment or removal, followed by T-ICD reimplantation (4). Extraction avoids the potential long-term risks of abandoned leads and allows magnetic resonance imaging to be performed. Indeed, although growing aggregate of data seems to question this (31), the presence of abandoned leads remains an absolute contraindication for magnetic resonance imaging. Nonetheless, in this series, the strategy of lead abandonment and S-ICD implantation avoided the possible complications associated with the extraction procedure, and appeared to be feasible and safe, with no significant increase in adverse outcomes in patients not requiring pacing. In current clinical practice (15), an S-ICD is preferred in younger patients and in those with a life expectancy longer than 10 years, who will probably survive their ICD leads (10). This may also apply to patients who experience ICD lead malfunction. Indeed, lead malfunction occurs most frequently in younger patients, both because they are more active and because a longer lead-dwelling time results in more prolonged lead stress (32). If the use of the S-ICD therefore appears justified in the event of lead malfunction, when pacing is not required, the actual need to extract malfunctioning leads may remain an open question, also in the light of the positive outcomes reported with the use of S-ICD after transvenous ICD extraction (17). In clinical practice, the risk profile of the patient, the number of leads, the time from the first implant are variables that may guide the management, as well as performing venography and discussing with patient before making a decision (Figure 3).

### Limitations

Our findings might be affected by a bias, owing to the retrospective study design. However, we included all consecutive patients who underwent S-ICD implantation after the abandonment of malfunctioning leads and all patients who underwent extraction and subsequent reimplantation of a single-chamber T-ICD in our analysis. The non-randomized comparison of the study represents an additional limitation. Indeed, a bias could derive from the differences between groups and specifically from factors influencing the operator's decision to extract or abandon a lead. In addition the small cohort size and the limited length of follow-up limit the statistical power and may have concealed differences between the groups. Indeed, a recent long-term analysis from a nationwide cohort study showed that the cumulative risk of interventions on abandoned ICD leads increased from 5.5% after 2.5 years to 15.2% after 10 years of abandonment (22). However our study represents the first experience of SICD implantation after abandonment of malfunctioning transvenous lead it and could pave the way for future larger studies.

### Conclusions

Although guidelines indicate the same class of recommendation both for lead abandonment and for removal followed by T-ICD reimplantation in the case of ICD lead malfunction, extraction is usually preferred in order to avoid the potential risks of abandoned leads. Nonetheless, in this study, the strategy of lead abandonment followed by S-ICD implantation proved feasible and safe, with no significant increase in adverse outcomes in patients who did not require pacing. This approach may constitute an option in selected clinical settings (e.g. high risk, failed extractions, etc.) in order to avoid the risks of lead removal. Longer follow-up studies are needed in order to fully understand the potential clinical value of this strategy.

## Data Availability Statement

The raw data supporting the conclusions of this article will be made available by the authors, without undue reservation.

## Ethics Statement

The studies involving human participants were reviewed and approved by Ethic Committee of Fondazione IRCCS POLICLINICO SAN MATTEO, Pavia, Italy. The patients/participants provided their written informed consent to participate in this study. Written informed consent was obtained from the individual(s) for the publication of any potentially identifiable images or data included in this article.

## Author Contributions

VR and SVi: concept/design and data collection. GT, GB, AD, PS, LO, GP, LC, and LS: data analysis/interpretation and data collection. FM, RR, and GN: drafting article. ML and SVa: critical revision of article. MGB, MB, and EB: approval of article. All authors contributed to the article and approved the submitted version.

## Conflict of Interest

ML and SV are employees of Boston Scientific. The remaining authors declare that the research was conducted in the absence of any commercial or financial relationships that could be construed as a potential conflict of interest.

## Publisher's Note

All claims expressed in this article are solely those of the authors and do not necessarily represent those of their affiliated organizations, or those of the publisher, the editors and the reviewers. Any product that may be evaluated in this article, or claim that may be made by its manufacturer, is not guaranteed or endorsed by the publisher.
